# Ginkgo Leaf Inspired Fabrication of Micro/Nanostructures and Demonstration of Flexible Enzyme-Free Glucose Sensors

**DOI:** 10.3390/s22197507

**Published:** 2022-10-03

**Authors:** Shulan Jiang, Yueqi Chen, Yong Peng

**Affiliations:** 1 School of Mechanical Engineering and Electronic Information, China University of Geosciences (Wuhan), Wuhan 430074, China; 2 Tribology Research Institute, School of Mechanical Engineering, Southwest Jiaotong University, Chengdu 610031, China

**Keywords:** ginkgo leaf, biofabrication, micro-nano hierarchical structure, flexible enzyme-free glucose sensor, electrochemical performance

## Abstract

Flexible enzyme-free glucose sensors have attracted widespread attention due to their importance and potential applications in clinical diagnosis, flexible wearable devices, and implanted devices in vivo. At present, there are still major problems in fabricating flexible enzyme-free glucose sensors with low detection limits, high stability, and high sensitivity at low cost, hindering their practical application. Here, we report a facile strategy for the fabrication of flexible non-enzymatic glucose sensors using ginkgo leaf as a template. NiO film and PEDOT:PSS composite film were deposited on the surface of the ginkgo leaf induced micro-nano hierarchical structure as a sensitive layer and a conductive layer, respectively. The as-prepared, flexible, enzyme-free glucose sensor exhibited excellent electrochemical performance toward glucose oxidation with a sensitivity of 0.7413 mA·mM^−1^/cm^−2^, an operating voltage of 0.55 V, a detection limit of 0.329 μM, and good anti-interference. Due to the simple fabrication process and performance reliability, the novel flexible enzyme-free glucose sensor is an attractive candidate for next generation wearable and implantable non-enzymatic glucose diagnostic devices.

## 1. Introduction

Diabetes, caused by insufficient insulin production or secretion, has steadily increased in prevalence in recent decades, and is a global public health problem [1,2,3]. Therefore, glucose detection or monitoring is becoming a greater priority in the analytical chemistry field. Much effort has been focused on developing suitable techniques for monitoring glucose levels with high sensitivity and reliability, fast response, good selectivity, and low cost [4,5]. There are now many approaches to measure glucose concentrations, such as optical techniques including infrared spectroscopy, Raman spectroscopy, and photo acoustic spectroscopy [6], as well as surface plasmon resonance biosensors [7], capacitive detection [8], electrochemiluminescence [9], colorimetry [10], the electrochemical method [11,12], and so forth. Among them, electrochemical sensors have attracted attention due to their high sensitivity, simple instrumentation, good reliability, and ease of operation [4,13].

To date, many enzyme-based electrochemical glucose sensors have shown high sensitivity, excellent selectivity, and practical feasibility. However, several drawbacks remain to be addressed, such as limited reproducibility and low stability during prolonged operation. The catalytic activity of enzymes can also be affected by pH, temperature, humidity, and toxic chemicals [14,15]. Non-enzymatic glucose sensors have become an alternative to enzymatic sensors. Currently, many nanomaterials, including noble metals (Pt, Ag, etc.) [16,17,18], transition metals (Cu, Co, etc.) [19,20], metal binary systems (Au-Pd, Ni-Co, etc.) [21,22], and metallic oxides (CuO, NiO, CoO, MnO_2_, etc.) are regarded as sensitive parts for non-enzymatic glucose sensors [23,24]. Among them, nickel oxide-based nanomaterials are intriguing and have been widely studied [25,26,27]. For example, Zhang et al. fabricated reduced graphene oxide/Ni(OH)_2_-based glucose sensors by the chemical suspension coating method [26]. Niu et al. reported 3D porous Ni networks for a non-enzymatic glucose sensor produced using a hydrogen-evolution-assisted electrodeposition strategy [4]. Most preparation processes have the problems of cumbersome multi-step fabrication processes and high preparation cost. Therefore, there is still an urgent need to develop a facile fabrication process of micro-nanostructures for high performance electrochemical glucose sensors. 

Inspired from the biofabrication strategy, the micro/nanostructures of natural plants can be used as an effective soft mold to fabricate micropatterned electrodes [28,29]. Because of its high elasticity, low cost, good adhesion, and good chemical inertness, polydimethylsiloxane (PDMS) is widely used in the preparation of micro-devices [30,31]. To obtain such a micro-nano hierarchical structure that exists in nature, using PDMS to directly replicate it is not only a simple preparation method but is also conducive to large-scale preparation. Therefore, in this research, ginkgo leaf was used as a template, and an advanced nanocasting manufacturing process was used to prepare a ginkgo bionic micro-nano hierarchical structure on the surface of PDMS. Subsequently, the Pt/Au film, PEDOT:PSS layer, and NiO film were deposited on the PDMS surface to obtain flexible enzyme-free glucose sensors. This method avoids cumbersome multi-step manufacturing processes and high preparation cost. The micro/nano hierarchical structures offer a large electrochemical surface area, increasing the rate of the transport of electrolytes through the solid/liquid interface due to the shortened diffusion length and beneficial diffusional regime. The electrocatalytic properties of the fabricated NiO electrode were systematically assessed. It was found that the sensor could provide ultrasensitive responses to glucose with favorable selectivity and excellent reliability.

## 2. Materials and Methods

### 2.1. Chemical and Materials

All chemicals of analytical grade or higher were used as received without further purification. Ultrapure water (18.2 MΩ cm) was used to prepare all aqueous solutions. PDMS (Sylgard 184, Down Corning Corp, Midland, MI, USA), composed of a silicone elastomer and a curing agent in a 10:1 wt ratio, was used. The components were thoroughly mixed and degassed in a vacuum to remove bubbles. Polystyrene sulfonate (PSS), 3,4-ethylenedioxythiophene (EDOT), nickel sulfate heptahydrate (NiSO_4_·7H_2_O), sulfuric acid (H_2_SO_4_), and hydrochloric acid (HCl) were purchased from Sinopharm.

### 2.2. Preparation of Flexible Enzyme-Free Glucose Sensor

**Figure 1:**
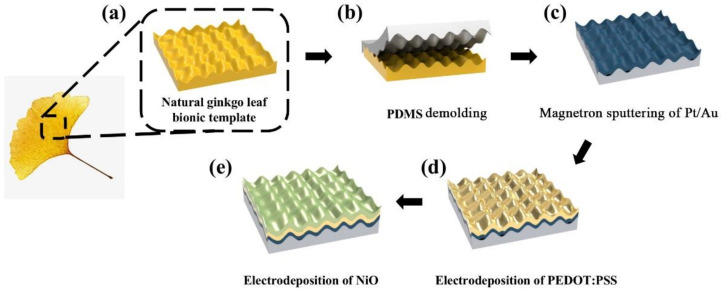
Schematic diagram for the preparation of a ginkgo leaf bionic micro-nano hierarchical structure.

Inspired by natural, bionic, micro-nano hierarchical structures existing in nature, this study proposes a flexible enzyme-free glucose sensor based on the ginkgo leaf bionic micro-nano hierarchical structure. The preparation process is shown in Figure 1. First, fresh ginkgo leaves were ultrasonically cleaned in alcohol and deionized water to remove impurities remaining on the surface, as shown in Figure 1a. Then, PDMS was spin-coated on the surface of the dried leaf samples and cured in an oven at 90 °C for 1 h. The PDMS-based micro-nano hierarchical structure was produced by peeling the PDMS from the ginkgo leaf, as shown in Figure 1b. Subsequently, a Pt/Au film was deposited on the surface by vapor deposition, as shown in Figure 1c. Prior to the PEDOT:PSS electrodeposition, 50 CV cycles were performed on all channels from −1.0 to 1.0 V versus a saturated calomel electrode (SCE) with a 1 V/s scanning rate in phosphate buffered saline (PBS, pH 7.4) as a cleaning step. The PEDOT:PSS composite was electrodeposited by the galvanostatic method in a three-electrode cell with the fabricated electrode sample as the working electrode, Pt mesh as a counter electrode, and SCE as a reference electrode, as shown in Figure 1d. The preparation of the electrolyte was as follows: 5 mg/mL PSS powder was added to 50 mL deionized water and stirred to dissolve. Then, 0.01 mol/L EDOT was added, and the solution was mixed uniformly using a stir bar for 2 h. Finally, NiO was electrodeposited on the sensor with a constant current density of 0.1 A/cm^2^ for 300 s by hydrogen evolution reaction in an electrolyte of 0.5 mol/L NiSO_4_·7H_2_O, 1.5 mol/L H_2_SO_4_, and 1 mol/L HCl. Thus, a flexible enzyme-free glucose sensor based on the bionic micro-nano hierarchical structure of ginkgo leaves was prepared, as shown in Figure 1e.

### 2.3. Measurements and Instrumentation

A field emission scanning electron microscope (SEM, JSM 7800F) was employed to characterize the structure and morphology of the sensor. An X-ray diffractometer (XRD, Empyrean) was employed to characterize the composition of active materials on the surface of the sensor. Electrochemical measurements were carried out on an electrochemical workstation (CHI660E, Shanghai, China) in a typical three-electrode configuration. The prepared flexible enzyme-free glucose sensor was used as the working electrode, the saturated calomel electrode or the Ag-AgCl electrode was used as the reference electrode, and a platinum electrode (10 × 10 mm) was used as the counter electrode. All measurements were performed at ambient temperatures.

## 3. Results and Discussion

**Figure 2:**
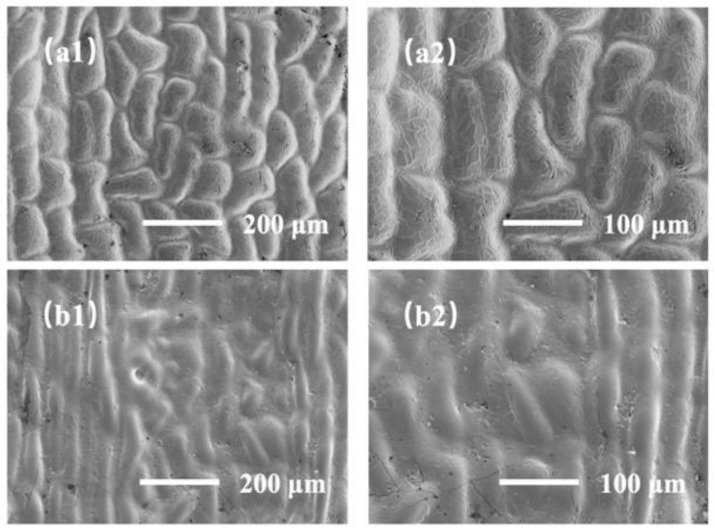
SEM images of demolded samples based on different templates at different magnifications. (**a1**–**a2**) mature leaves (yellow), (**b1**–**b2**) immature leaves (green).

In order to obtain the demolding samples of ginkgo leaf with the largest specific surface areas, this experiment compared the morphological differences of the demolding structures of the mature leaf (yellow) and immature leaf (green) samples, as shown in Figure 2. Figure 2a1,a2 show the surface morphology of the mature ginkgo leaf samples after demolding at 300× and 500× magnification, respectively. Figure 2b1,b2 show the surface morphology of the immature leaf samples after demolding at 300× and 500× magnification, respectively. By comparing Figure 2a,b, we can see that the micro/nano hierarchical structure of the mature blades is more obvious. Therefore, the specific surface area of the demolded sample of the mature blade was larger, making it more suitable for the supporting layer of the sensor.

**Figure 3:**
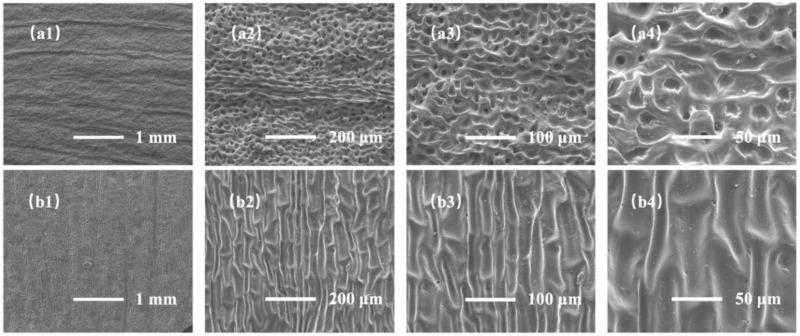
SEM images of demolded samples from both sides of a ginkgo leaf at different magnifications. (**a1**–**a4**) back side; (**b1**–**b4**) front side.

At the same time, we explored the morphological differences between the demolding structure from the back and the front of ginkgo leaf, as shown in Figure 3. SEM images of the demolded samples from the back of the ginkgo leaf are shown in Figure 3a1–a4. The demolded samples mainly showed micron-level linear structures and pore structures. The structural pore size was 5–20 μm. Figure 3b1–b4 show SEM images of the demolded samples from the front of the ginkgo leaf. The demolded samples mainly showed micron-level pit structures and nano-scale fold structures; the pit size was 15–30 μm. It is not difficult to see that the sample demolded from the back of the ginkgo leaf had a larger specific surface area, and the nano-scale folds were more conducive to the insertion and extraction of ions.

In addition, we found that the nano-scale wrinkles produced on the demolding structure were mainly caused by the dehydration of the ginkgo leaves during demolding. Dehydration would affect the adhesion between the PDMS surface and the ginkgo leaves, so different demolding parameters have a certain influence on the morphology of the demolding structure. Considering that there were differences in the levels of maturity of different ginkgo leaves, when exploring the influence of curing temperature and time in this experiment, we selected similar areas of the same leaf and divided them evenly into multiple parts.

**Figure 4:**
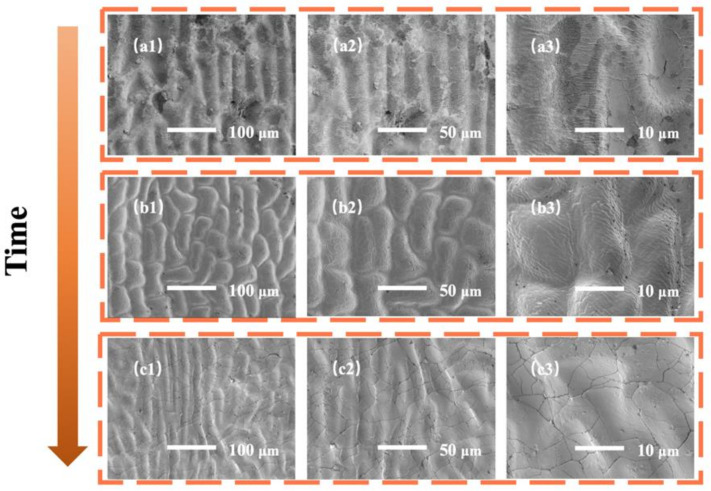
SEM images of demolded samples of ginkgo leaves micro/nano structure under different curing times at different magnifications. (**a1**–**a3**) 60 min, (**b1**–**b3**) 90 min, (**c1**–**c3**) 120 min.

Figure 4 shows the surface morphology of the ginkgo leaf bionic micro-nano structure demolded samples with different curing times (60 min, 90 min, and 120 min). The results showed that when the curing time was 60 min, the PDMS just reached the solidified state, and there were intermittent nano-scale folds on the surface of the crater structure of the demolding sample, as shown in Figure 4a1–a3. When the curing time was increased to 90 min, the nano-level folds on the surface of the pit structure were dense and relatively uniform, as shown in Figure 4b1–b3. When the curing time was further increased to 120 min, the surface of the pit structure was mainly crack-like, as shown in Figure 4c1–c3. This is probably caused by different degree of dehydration of ginkgo leaves at different curing times. The ginkgo leaves gradually dehydrated and dense and uniform microfold structures appeared with increasing curing time. With a further increase of curing time, the dehydration of ginkgo leaves caused severe cracks of the surface structure. Based on the above discussion and analysis, this study chose 90 min as the best curing time for the demolding of the ginkgo leaf bionic micro-nano structure. 

**Figure 5:**
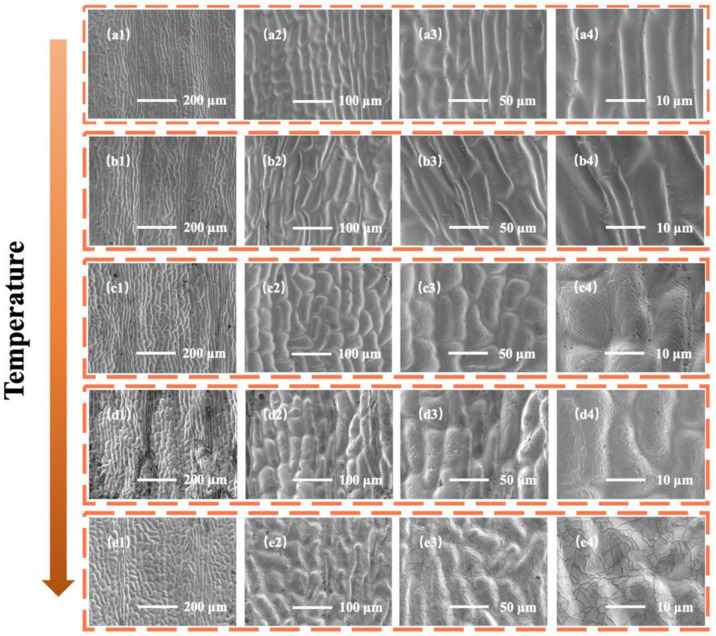
SEM images of bionic micro-nano structure samples of ginkgo leaf under different curing temperatures at different magnifications. (**a1**–**a4**) 70 °C, (**b1**–**b4**) 90 °C, (**c1**–**c4**) 110 °C, (**d1**–**d4**) 130 °C and (**e1**–**e4**) 150 °C.

The surface morphologies of ginkgo leaf demolded samples at different curing temperatures (70 °C, 90 °C, 110 °C, 130 °C, and 150 °C) are shown in Figure 5. The surface of the demolded sample only had micron-level pit structures when the temperature was 70 °C. As the curing temperature increased, some nano-scale wrinkles appeared (90 °C), becoming dense and uniform at 110 °C. With a further increase of curing temperature, the nano-scale folds on the surface of the pit structure became stacked (130 °C) and gradually disappeared to form cracks (150 °C). This phenomenon may have been due to the different dehydration conditions of ginkgo leaves at different curing temperatures. The ginkgo leaves gradually dehydrated and dense and uniform nano-scale wrinkle structures appeared with increasing curing temperatures. With the further increase of curing temperature, the dehydration of ginkgo leaves gave rise to severe cracks of the surface structure. Based on the above discussion and analysis, this study chose 110 °C as the best curing temperature for the demolding of the ginkgo leaf bionic micro-nano structure.

**Figure 6:**
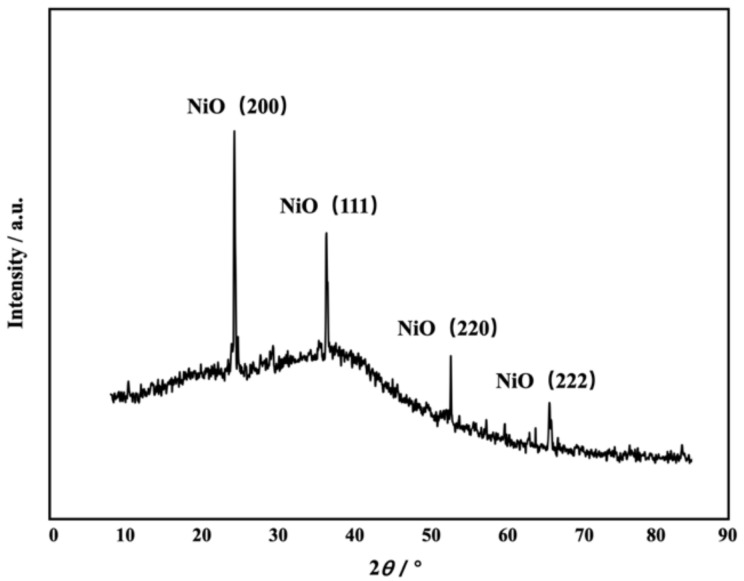
X-ray diffraction pattern of the NiO thin film deposited onto a bionic micro-nano structure substrate.

The composition of the active material on the sensor surface was characterized by XRD. As shown in Figure 6, four diffraction peaks, at 24.2°, 38.4°, 51.5°, and 67.2°, were attributed to the (200), (111), (220) and (222) crystalline planes, respectively [1]. The X-ray diffraction results showed that the active material, i.e., NiO, on the sensor surface was of high purity, ensuring the accuracy of subsequent tests [2].

**Figure 7:**
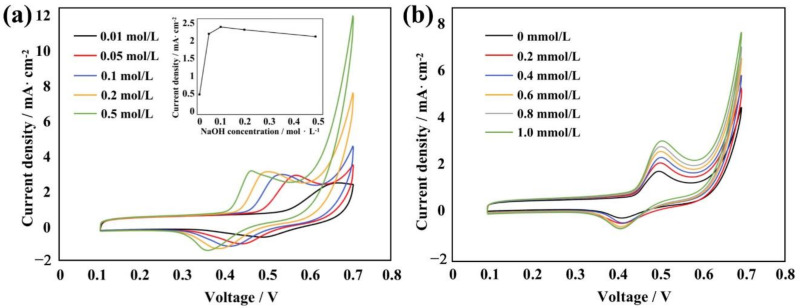
(**a**) Cyclic voltammetry curves of the prepared NiO electrode at different concentrations of NaOH. (**b**) Cyclic voltammetry curves of the prepared NiO electrode at different concentrations of glucose.

To demonstrate the application of the micro/nanostructures, NiO was integrated into the micro/nanostructures for our enzyme-free glucose sensor. The cyclic voltametric performance of the plane PDMS and PDMS micro/nanostructures was tested; the results (Figure S1) showed the PDMS micro/nanostructures exhibited better electrochemical performance. We also conducted CV experiments in NaOH and glucose solution using the fabricated NiO electrode, respectively. Figure 7a shows the cyclic voltammograms recorded in the NaOH solution for the NiO electrode. As the concentration of the NaOH solution increased, the oxidation and reduction peaks on the curve moved in a negative direction, but the shape of the curve remained basically unchanged. This indicated that the generation of a redox peak was related to the concentration of OH-. At the same time, the current response of different concentrations of NaOH solution under the same concentration of glucose was further studied. The results showeds that when the concentration of the NaOH solution was 0.1 mol/L, the current response was most obvious, as shown in Figure 7a.

Figure 7b shows the cyclic voltammograms recorded in the glucose solution for the NiO electrode. The scan rate was 100 mV/s. As the concentration of the glucose solution increased, the shape of the curve did not show any obvious changes but the oxidation and reduction peaks both increased significantly; the change of the former was greater than that of the latter. This indicated that the conversion rate of Ni^2+^ to Ni^3+^ and the catalytic oxidation rate of glucose by the sensor was gradually increasing. Therefore, the sensor had a significant catalytic oxidation effect on glucose. As the glucose concentration increased, the reaction rate gradually increased, and the detection effect was better. 

The electrochemical behavior in the presence of glucose can be explained by the following reaction scheme: First, NiO on the sensor surface was converted into hydrated α-Ni(OH)_2_ and anhydrous β-Ni(OH)_2_ in the alkaline electrolyte, as shown in Equation (1) [1]. Next, when a cyclic voltage was applied to the sensor, α-Ni(OH)_2_ and β- Ni(OH)_2_ combined with OH- in the electrolyte and were converted into their respective oxyhydroxide species, γ-NiOOH and β-NiOOH, according to Equation (2) [3]. Finally, when glucose was added, a catalytic reaction occurred, i.e., the oxidation of glucose to glucolactone, as shown in Equation (3) [2].

(1) $${\mathrm{NiO}+H}_{2}O\;\to \;{\mathrm{Ni}(\mathrm{OH})}_{2}$$

(2) $${\mathrm{Ni}(\mathrm{OH})}_{2}+{\mathrm{OH}}^{-}\;\to {\;\mathrm{NiOOH}+H}_{2}O+e^{-}$$

(3) $$\mathrm{NiOOH}+\mathrm{glucose}\;\to {\;\mathrm{Ni}(\mathrm{OH})}_{2}+\mathrm{glucolactone}$$

**Figure 8:**
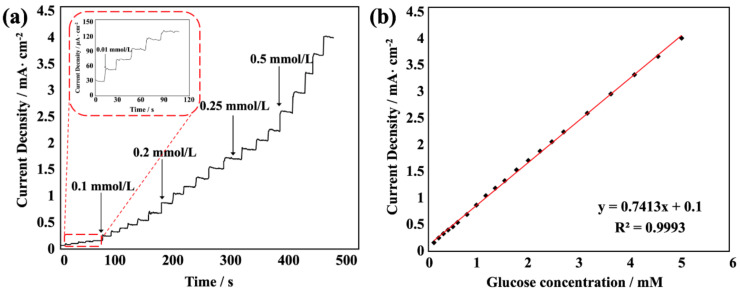
(**a**) Typical amperometric current response of the flexible enzyme-free glucose sensor upon the successive injection of glucose at different concentrations. (**b**) Plot of electrocatalytic current of glucose vs. the corresponding concentration.

Figure 8 shows a typical amperometric response of the flexible enzyme-free glucose sensor upon the successive addition of a certain concentration of glucose to 0.1 mol/L NaOH at the optimal potential of 0.52 V (at which the electrode response was most obvious and stable in our experiment). As the concentration of glucose solution increased, the current intensity response of the sensor increased linearly. According to the glucose concentration and current intensity response value, the fitted linear equation is y = 0.9773x + 0.1465, R^2^ = 0.9979. The results show that the sensitivity of the sensor was 0.9773 mA·mM^−1^·cm^−2^, and the detection limit could reach values as low as 0.371 μM, based on a signal-to-noise ratio of 3. According to the literature, Ni(OH)_2_ and nanostructures, as well as NiO nanorod-based glucose sensor electrodes, showed limits of detection of 70 μM and 8.1 μM, respectively, with a sensitivity of 12.09 and 24.0 μA·mM^−1^·cm^−2^ [27]. A porous NiO nanofilm electrode also showed a detection limit of 0.34 μM and a sensitivity of 1.68 mA·mM^−1^·cm^−2^ [32]. A glucose oxidase enzyme entrapped in PEDOT:PSS conductive polymer with a silicon carbide nanoparticle, electrospun, nanofibrous membrane-based glucose sensing electrode showed a sensitivity of 30.75 μA·mM^−1^ cm^−2^ and a detection limit was 0.56 μM [4]. By comparison, our glucose sensor has good sensitivity and a low detection limit, and has certain advantages for the detection of glucose.

**Figure 9:**
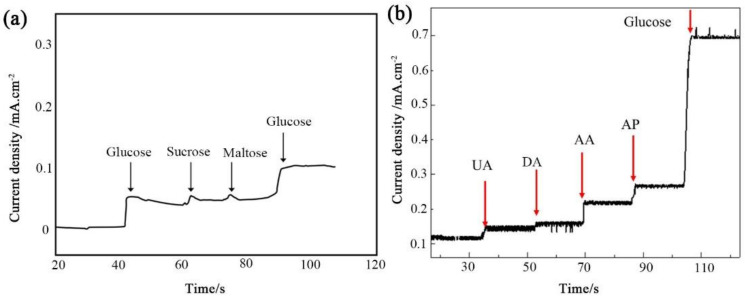
(**a**) Amperometric response of the NiO integrated micro/nanostructures electrode upon the addition of glucose, sucrose, and maltose. (**b**) Amperometric response of the electrode to different interference species, i.e., UA, DA, AA, AP, and glucose, respectively.

Anti-interference was also a criterion to evaluate the electrochemical performance for practical applications. Thus, it was essential to investigate the selectivity of the flexible enzyme-free glucose sensor. An interference experiment was performed, measuring the amperometric response to the addition of 0.05 mmol/L glucose, sucrose, and maltose in 0.1 mol/L NaOH alkaline medium at a potential of +0.52 V (vs. Ag/AgCl), respectively. As shown in Figure 9a, doubling the addition of glucose both caused a strong current response. The addition of the same concentrations of sucrose and maltose produced negligible responses. Moreover, the interference species of uric acid (UA), ascorbic acid (AA), dopamine (DA), and 4-acetamino phenol (AP) were tested by the addition of 0.5 mmol/L glucose and 0.05 mmol/L interference species in 0.1 mol/L NaOH at a bias of +0.52 V (shown in Figure 9b). The results confirmed the good anti-interference performance of our flexible sensor. In addition, the micro/nanostructure glucose sensor electrode showed good stability after repeated CV cycling tests.

This study proposed a facile fabrication method for micro/nanostructures, and the fabrication parameters have been discussed. The micro/nanostructures were then integrated with NiO nanostructures. This paper reports a preliminary exploration and demonstration of the fabricated micro/nanostructures for glucose sensors. Further study of the glucose sensor in PBS buffer or serum or the demonstration of the response of a micro/nanostructure-based electrode to other active substances will be conducted in the future.

## 4. Conclusions

In summary, a facile strategy was developed to fabricate a flexible NiO electrode with a micro-nano hierarchical structure for use in flexible, non-enzymatic, electrochemical amperometric sensors of glucose concentrations. The characteristics of the micro-nano hierarchical structures of the pristine surface of ginkgo leaf were first analyzed. The effects of fabrication parameters such as the type of ginkgo leaf, the mold release temperature, and the time on the PDMS micro-nano hierarchical structure were studied. Based on this, the best preparation parameters were used to prepare micro-nano hierarchical structures on the surface of PDMS by demolding. A Pt/Au/PEDOT:PSS film, serving as a conductive layer, was integrated on the surface of the fabricated micro-nano hierarchical structure. NiO was further deposited on the surface of the sample, acting as reaction layer for the sensor. This NiO electrode with a micro-nano hierarchical structure possessed high catalytic activity, a large accessible surface area, and good conductivity. The obtained flexible enzyme-free glucose sensor exhibited a number of excellent features, such as high sensitivity, high linearity, low operating voltage, low detection limit, and good anti-interference. It is believed that the flexible enzyme-free glucose sensor will be widely used in highly sensitive enzyme-free glucose sensors or wearable flexible electronic devices. 

## Data Availability

The data presented in this study are available on request from the corresponding author.

## Supplementary Materials

The following supporting information can be downloaded at: https://www.mdpi.com/article/10.3390/s22197507/s1, Figure S1. CV comparison of the plane PDMS and PDMS micro/nanostructures, which were tested in 0.1 mol/L NaOH solution at the scan rate of 100 mV/s.

## References

[1] Zhao J., Wei L., Peng C., Su Y., Yang Z., Zhang L., Wei H., Zhang Y. (2013). A non-enzymatic glucose sensor based on the composite of cubic Cu nanoparticles and arc-synthesized multi-walled carbon nanotubes. Biosens. Bioelectron..

[2] Yoon H., Xuan X., Jeong S., Park J.Y. (2018). Wearable, robust, non-enzymatic continuous glucose monitoring system and its in vivo investigation. Biosens. Bioelectron..

[3] Hwang D.W., Lee S., Seo M., Chung T.D. (2018). Recent advances in electrochemical non-enzymatic glucose sensors—A review. Anal. Chim. ACTA.

[4] Niu X., Lan M., Zhao H., Chen C. (2013). Highly sensitive and selective nonenzymatic detection of glucose using Three-Dimensional porous nickel nanostructures. Anal. Chem..

[5] Qin X., Lu W., Luo Y., Chang G., Asiri A.M., Al-Youbi A.O., Sun X. (2012). Synthesis of Ag nanoparticle-decorated 2,4,6-tris(2-pyridyl)-1,3,5-triazine nanobelts and their application for H_2_O_2_ and glucose detection. Analyst.

[6] Barone P.W., Parker R.S., Strano M.S. (2005). In vivo fluorescence detection of glucose using a single-walled carbon nanotube optical sensor:  Design, Fluorophore properties, Advantages, and Disadvantages. Anal. Chem..

[7] Shen X.W., Huang C.Z., Li Y.F. (2007). Localized surface plasmon resonance sensing detection of glucose in the serum samples of diabetes sufferers based on the redox reaction of chlorauric acid. Talanta.

[8] Cheng Z., Wang E., Yang X. (2001). Capacitive detection of glucose using molecularly imprinted polymers. Biosens. Bioelectron..

[9] Kremeskotter J., Wilson R., Schiffrin D.J., Luff B.J., Wilkinson J.S. (1995). Detection of glucose via electrochemiluminescence in a thin-layer cell with a planar optical waveguide. Meas. Sci. Technol..

[10] Morikawa M.-a., Kimizuka N., Yoshihara M., Endo T. (2002). New colorimetric detection of glucose by means of electron-accepting indicators: Ligand substitution of [Fe(acac)3−n(phen)n]n+ complexes triggered by electron transfer from glucose oxidase. Chem. Eur. J..

[11] Kim Y.J., Chinnadayyala S.R., Le H.T., Cho S. (2022). Sensitive electrochemical non-enzymatic detection of glucose based wireless data transmission. Sensors.

[12] Adeel M., Rahman M.M., Caligiuri I., Canzonieri V., Rizzolio F., Daniele S. (2020). Recent advances of electrochemical and optical enzyme-free glucose sensors operating at physiological conditions. Biosens. Bioelectron..

[13] Ding J., Li X., Zhou L., Yang R., Yan F., Su B. (2020). Electrodeposition of nickel nanostructures using silica nanochannels as confinement for low-fouling enzyme-free glucose detection. J. Mater. Chem. B.

[14] Zhang Y., Li N., Xiang Y., Wang D., Zhang P., Wang Y., Lu S., Xu R., Zhao J. (2020). A flexible non-enzymatic glucose sensor based on copper nanoparticles anchored on laser-induced graphene. Carbon.

[15] Hassan M.H., Vyas C., Grieve B., Bartolo P. (2021). Recent advances in enzymatic and non-enzymatic electrochemical glucose sensing. Sensors.

[16] Luo Y., Kong F.-Y., Li C., Shi J.-J., Lv W.-X., Wang W. (2016). One-pot preparation of reduced graphene oxide-carbon nanotube decorated with Au nanoparticles based on protein for non-enzymatic electrochemical sensing of glucose. Sens. Actuators B Chem..

[17] Baghayeri M., Amiri A., Farhadi S. (2016). Development of non-enzymatic glucose sensor based on efficient loading Ag nanoparticles on functionalized carbon nanotubes. Sens. Actuators B Chem..

[18] Baghayeri M., Amiri A., Motamedifar A. (2016). Investigation about electrocatalytic oxidation of glucose on loaded Ag nanoparticles on functionalized carbon nanotubes. Ionics.

[19] Gao W., Li Q., Dou M., Zhang Z., Wang F. (2018). Self-supported Ni nanoparticles embedded on nitrogen-doped carbon derived from nickel polyphthalocyanine for high-performance non-enzymatic glucose detection. J. Mater. Chem. B.

[20] Li Y., Tang X., Shen Z., Dong J. (2019). Prediction of total volatile basic nitrogen (TVB-N) content of chilled beef for freshness evaluation by using viscoelasticity based on airflow and laser technique. Food Chem..

[21] Li X., Du X. (2017). Molybdenum disulfide nanosheets supported Au-Pd bimetallic nanoparticles for non-enzymatic electrochemical sensing of hydrogen peroxide and glucose. Sens. Actuators B Chem..

[22] Ramachandran K., Raj kumar T., Babu K.J., Gnana kumar G. (2016). Ni-Co bimetal nanowires filled multiwalled carbon nanotubes for the highly sensitive and selective non-enzymatic glucose sensor applications. Sci. Rep..

[23] Ponnusamy R., Venkatesan R., Kandasamy M., Chakraborty B., Rout C.S. (2019). MnO_2_ polymorph selection for non-enzymatic glucose detection: An integrated experimental and density functional theory investigation. Appl. Surf. Sci..

[24] Baghayeri M., Sedrpoushan A., Mohammadi A., Heidari M. (2017). A non-enzymatic glucose sensor based on NiO nanoparticles/functionalized SBA 15/MWCNT-modified carbon paste electrode. Ionics.

[25] Darvishi S., Souissi M., Karimzadeh F., Kharaziha M., Sahara R., Ahadian S. (2017). Ni nanoparticle-decorated reduced graphene oxide for non-enzymatic glucose sensing: An experimental and modeling study. Electrochim. Acta.

[26] Zhang Y., Xu F., Sun Y., Shi Y., Wen Z., Li Z. (2011). Assembly of Ni(OH)_2_ nanoplates on reduced graphene oxide: A two dimensional nanocomposite for enzyme-free glucose sensing. J. Mater. Chem..

[27] Pal N., Banerjee S., Bhaumik A. (2018). A facile route for the syntheses of Ni(OH)_2_ and NiO nanostructures as potential candidates for non-enzymatic glucose sensor. J. Colloid Interface Sci..

[28] Seol M.-L., Woo J.-H., Lee D.-I., Im H., Hur J., Choi Y.-K. (2014). Nature-replicated nano-in-micro structures for triboelectric energy harvesting. Small.

[29] Kong T., Luo G., Zhao Y., Liu Z. (2019). Bioinspired superwettability micro/nanoarchitectures: Fabrications and applications. Adv. Funct. Mater..

[30] Wang Z., Guo S., Li H., Wang B., Sun Y., Xu Z., Chen X., Wu K., Zhang X., Xing F. (2018). The semiconductor/conductor interface piezoresistive effect in an organic transistor for highly sensitive pressure sensors. Adv. Mater..

[31] Tang X., Wu C., Gan L., Zhang T., Zhou T., Huang J., Wang H., Xie C., Zeng D. (2019). Multilevel microstructured flexible pressure sensors with ultrahigh sensitivity and ultrawide pressure range for versatile electronic skins. Small.

[32] Garcia-Garcia F.J., Salazar P., Yubero F., González-Elipe A.R. (2016). Non-enzymatic glucose electrochemical sensor made of porous NiO thin films prepared by reactive magnetron sputtering at oblique angles. Electrochim. Acta.

