# Supplementary material for: Ginkgo Leaf Inspired Fabrication of Micro/Nanostructures and Demonstration of Flexible Enzyme-Free Glucose Sensors
Source: Sensors (Basel). 2022 Oct 3;22(19):7507. doi: 10.3390/s22197507 (PMC9571730; doi:10.3390/s22197507)
Supplement: Supplementary file 1 [file sensors-22-07507-s001.zip › sensors-1902760-supplementary.pdf]

Supporting Information

Article

Ginkgo leaf inspired fabrication of micro/nanostructures and the demonstration of flexible enzyme-free glucose sensors

Shulan Jiang[1*], Yueqi Chen[2], Yong Peng[2]

1 School of Mechanical Engineering and Electronic Information, China University of Geosciences (Wuhan), Wuhan 430074, China
2 Tribology Research Institute, School of Mechanical Engineering, Southwest Jiaotong University, Chengdu 610031, China
* Correspondence: jiangshulan@cug.edu.cn

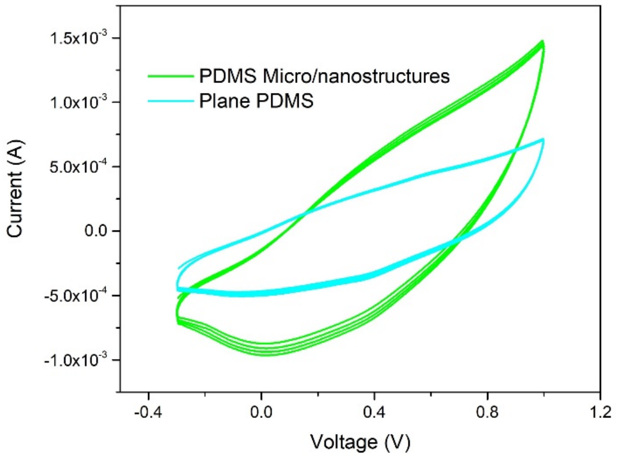

Figure S1. CV comparison of the plane PDMS and PDMS micro/nanostructures, which were tested in 0.1mol/L NaOH solution at the scan rate of 100 mV/s.
